# Differential diagnosis of testicular embryonal rhabdomyosarcoma and testicular seminoma with enhanced CT: a retrospective study

**DOI:** 10.3389/fonc.2026.1707887

**Published:** 2026-06-03

**Authors:** Zhixu Zhang, Pan Liang, Songwei Yue, Yonggao Zhang, Hongyan Zhang, Zhigang Zhou, Jianbo Gao

**Affiliations:** 1 Departments of Radiology, the First Affiliated Hospital of Zhengzhou University, Zhengzhou, China; 2 Departments of Pathology, the First Affiliated Hospital of Zhengzhou University, Zhengzhou, China

**Keywords:** diagnosis, difference, testicular embryonal rhabdomyosarcoma, testicular seminoma, tomography, X-ray computed

## Abstract

**Background:**

Testicular embryonal rhabdomyosarcoma (ERMS) is a rare type of testicular neoplasm. This study aimed to conduct a differential diagnosis between testicular seminoma and testicular ERMS based on enhanced CT features.

**Methods:**

In this retrospective study, 15 cases of intratesticular ERMS that were pathologically confirmed and 30 cases of testicular seminoma were collected from December 2012 to May 2024. The general information of the patients was collected, and CT images were retrieved, reviewed, and reanalyzed. CT parameters were measured (the length and short diameter of each lesion, as well as the CT attenuation in the non-enhanced phase, arterial phase, and venous phase), as well as laboratory indicators such as alpha-fetoprotein (AFP), lactate dehydrogenase (LDH), and β-human chorionic gonadotropin (β-HCG). Statistical analysis for quantitative data was conducted using an independent sample t-test or a non-parametric test, while the chi-square test was used for qualitative parameters. Further, a receiver operating characteristic (ROC) curve analysis was conducted to test the diagnostic value for differential diagnosis. The existence of postoperative disease progression (recurrence or metastasis) in the two groups was compared using the chi-square test.

**Results:**

A total of 45 patients were involved in the study (15 intratesticular ERMS and 30 testicular seminoma). There were differences in age, CT parameters, and laboratory indicators between rhabdomyosarcoma and seminoma (all *p* < 0.05). Similarly, the parallel relationship between the lesion and the ipsilateral inguinal region, as well as the vascular ball sign and other signs, also showed statistically significant differences (all *p* < 0.05). Further single and multi-factor analyses revealed that the parallel relationship between the lesion and the ipsilateral inguinal region, as well as the vascular ball sign, showed statistically significant differences (*p* < 0.05). The area under the ROC curve (AUC) of the model by logistic regression reached 0.961 [95% confidence interval (CI), 0.857–0.996], with a sensitivity of 86.7% and a specificity of 96.7%. Furthermore, the probability of postoperative disease progression of testicular ERMS is significantly higher than that of testicular seminoma (66.67% vs. 3.3%). Among the 15 ERMS patients, 10 cases (66.7%) had postoperative disease progression, including five cases of recurrence and five cases of distant metastasis. The median time to progression was 7 months (range 2–16.5 months).

**Conclusions:**

The existence of a parallel relationship between the lesion and the ipsilateral inguinal region, as well as the vascular ball sign, is helpful in differentiating testicular ERMS from testicular seminoma.

## Introduction

Rhabdomyosarcoma (RMS) is a rare type of malignant neoplasm that originates from the precursor cells of muscle formation, which mainly occurs in children and adolescents (1). Embryonal rhabdomyosarcoma (ERMS) is the most common subtype among the four types of rhabdomyosarcoma (2). It is extremely rare to occur in the testis and its surroundings (derived from the epididymis, distal end of the spermatic cord, or the tunica albuginea of the testis) (3). ERMS located beside the testis accounts for only 7% of all RMS cases, and it is even rarer when it occurs within the testis (4). ERMS within the testicles is a rare and aggressive neoplasm that progresses rapidly and has a poor prognosis (5). Most of the previous literature consists of case reports, and the clinical features of this disease are not specific (6). According to the literature, due to the rarity of this disease, the treatment strategy is based on the treatment measures for non-seminoma of the testicles (7, 8). Early identification of this lesion and active treatment are very important for improving the prognosis of patients.

Testicular seminoma is the most common malignant neoplasm within the testicles, accounting for 55% to 60% of all germ cell neoplasms in the testicles (9, 10). The treatment for testicular seminoma mainly involves surgical removal and chemotherapy (8–10). Although 90% of patients have a good prognosis, this neoplasm mainly spreads through lymphatic metastasis (11). Thus, early and standardized treatment can significantly reduce the risk of metastasis. Approximately 80% of testicular seminoma cases are confined to the testis at the time of diagnosis. Without adjuvant treatment, the estimated recurrence rate is 13% to 20% (12). Due to the differences in treatment strategies, seminoma and non-seminoma have always been the focus and challenge in clinical practice. Currently, the gold standard for diagnosing testicular neoplasms relies on histopathological examination, and early preoperative diagnosis is particularly important.

Whether it is testicular ERMS or seminoma, there are no significant differences in symptoms and signs. Both present as painless masses in the testicle, which are hard upon palpation (13, 14). Ultrasound, as a commonly used diagnostic method, not only can determine whether the mass is located within or beside the testicle but also can be combined with color Doppler to observe the blood supply of the mass (8). As the preferred imaging examination method, CT not only enables the display of the primary lesion but also allows for the observation of lymph nodes and distant metastases (8).

Primary intratesticular ERMS is extremely rare; most of the existing literature on intratesticular ERMS in the testis consists of case reports, and the imaging features have not been fully reported (6). In this study, we retrospectively analyzed the CT morphology and enhancement features of intratesticular ERMS and testicular seminoma in the testis to deepen our understanding of how to distinguish between them.

## Materials and methods

This retrospective study was approved by the Institutional Review Board of the First Affiliated Hospital of Zhengzhou University (No. 2022-KY-1447-002). Since it was a retrospective study, the patients’ informed consent was waived.

### Patients

A total of 18 cases of testicular embryonal rhabdomyosarcoma and 30 cases of testicular seminoma diagnosed by surgical pathology at the First Affiliated Hospital of Zhengzhou University from December 2012 to May 2024 were retrospectively collected. All cases of ERMS were pathologically confirmed as intratesticular. The 30 testicular seminoma cases were randomly enrolled from all eligible cases in the same hospital during the same period (from December 2012 to May 2024) using a computer-generated random number table, and they were within the same research period as the ERMS cases. All patients underwent enhanced CT scans after admission and before surgery. The inclusion criteria were A) pathological diagnosis of embryonal rhabdomyosarcoma or testicular seminoma, B) only located within the testicle, and C) complete clinical data. The exclusion criteria were A) inability to obtain data or having incomplete imaging data, B) not undergoing surgery, and C) incomplete follow-up data. Finally, 45 patients were included, including 15 testicular ERMS and 30 testicular seminoma.

### Data collection

We collected the following information: 1) clinicopathological characteristics, including variables such as age and cryptorchidism; 2) the quantitative parameters of the CT features of the lesion, including its length, short diameter, and the CT attenuation in the non-enhanced phase, arterial phase, and venous phase; 3) biochemical indicators, including alpha-fetoprotein (AFP), carcinoembryonic antigen (CEA), carbohydrate antigen 125 (CA125), Carbohydrate antigen 19-9 (CA19-9), lactate dehydrogenase (LDH), and β-human chorionic gonadotropin (β-HCG); and 5) the qualitative parameters of the CT features of the lesion, including the lesion site (left side and right side), the parallel relationship between the lesion and the ipsilateral inguinal region, the vascular ball sign, abnormal widening of the spermatic veins, and disease components (solid and cystic-solid). Please refer to the **Supplementary Material** for detailed instructions.

### Image acquisition and analysis

Within 30 days after each patient received treatment, they all underwent advanced CT scans of the abdomen (including the pelvic area) or only pelvic CT scans, with the scanning range covering the entire pelvic floor, including the testicular lesions. As this study was retrospective, it involved multiple CT scanning devices. Please refer to the **Supplementary Material** for detailed instructions.

Two experienced radiology diagnostic physicians with over 10 years of experience jointly reviewed the films and analyzed the CT images while blinded to the clinicopathological information, and the CT images were interpreted independently. They collected and recorded the quantitative and qualitative parameters related to CT. The quantitative parameters of the CT features of the lesion included its length, short diameter, and the CT attenuation in the non-enhanced phase, arterial phase, and venous phase. All the CT attenuation, short diameter, and length data were independently measured three times by two radiological diagnostic physicians. Then, the mean values of the two radiological diagnostic physicians were calculated for the next statistical analysis.

The qualitative parameters of the CT features of the lesion included the lesion site (left side and right side), the parallel relationship between the lesion and the ipsilateral inguinal region, the vascular ball sign, abnormal widening of the spermatic veins, disease components (solid and cystic-solid), abnormal dilation of the testicular artery, lymph node metastasis, internal necrosis within the lesion, abnormality of blood vessels within the lesion, and cryptorchidism.

The electronic medical record system was adopted, and telephone follow-up was conducted to monitor the patients’ conditions in order to determine whether there was disease progression (recurrence or metastasis) after the surgery; the progression of the disease was defined as the endpoint event. Disease progression was strictly defined as pathologically confirmed recurrence or distant metastasis.

For qualitative parameters, if there were differences in the results, then the decision would be made after a joint discussion by two radiologists. Please refer to the **Supplementary Material** for detailed instructions.

### Statistical analysis

Based on the statistical analysis conducted using the SPSS software (version 21.0, IBM), the difference was statistically significant (*p* < 0.05). Continuous variables (quantitative parameters) were normalized using the Kolmogorov–Smirnov test in univariate analysis, and normal distribution data were expressed as mean ± standard deviation (SD). The differences in continuous variables (quantitative parameters) were compared using the Mann–Whitney *U* test or the independent sample t-test. Comparison of unordered categorical variables (qualitative parameters) was performed using the chi-square test or Fisher’s exact probability method. Inter-observer agreement for the parallel relationship was evaluated using the kappa coefficient. Variance inflation factor (VIF) was used for conducting collinearity analysis. Multivariate logistic regression analysis was used for variables with statistically significant differences to obtain the independent predictive factors for differentiating testicular ERMS and testicular seminoma, and a combined model was established. The diagnostic efficacy of single parameters and the combined parameter was evaluated using the receiver operating characteristic (ROC) curve, along with its area under the ROC curve [AUC and 95% confidence interval (CI)], Youden’s index, sensitivity, and specificity. An AUC exceeding 0.70 was deemed satisfactory. The AUC was compared using DeLong’s test. Internal validation was performed using bootstrap resampling with 1,000 iterations to obtain the bootstrap-corrected AUC and assess model stability. Statistical significance was set at the level of *p* < 0.05.

## Results

### Clinical characteristics

This study ultimately included 15 testicular ERMS and 30 testicular seminoma cases. The age range for the patients with testicular ERMS was 1–22 years. The age range for the patients with testicular seminoma was 18–57 years.

**Table 1** summarizes the clinical characteristics and CT quantitative parameters between the two groups of testicular ERMS and testicular seminoma. Between the two groups, there were significant differences in age, AFP, CA125, CA19-9, LDH, β-HCG, lesion length (L_length), lesion short diameter (L_short diameter), CT attenuation of lesion unenhanced phase (L_ctp), CT attenuation of lesion arterial phase (L_cta), and CT attenuation of lesion venous phase (L_ctv) (all *p* < 0.05).

**Table 1:** Clinical characteristics and CT quantitative parameters.

Characteristic	Testicular seminoma (*n* = 30)	Testicular ERMS (*n* = 15)	*T*/*z*	*P*
Age, years	32.00 (28.00, 35.00)	13.00 (7.00, 17.00)	−5.326	0.000**
AFP, ng/mL	1.87 (1.51, 3.10)	0.96 (0.90, 1.70)	−2.698	0.007**
CEA, ng/mL	1.575 (1.0, 2.0)	1.31 (0.60, 1.80)	−0.987	0.323
CA125, U/mL	6.35 (4.31, 8.43)	14.06 (10.30, 23.42)	−4.094	0.000**
CA19-9, U/mL	4.44 (3.20, 7.70)	7.19 (5.00, 14.80)	−2.131	0.033*
LDH, u/L	214.00 (191.00, 237.00)	320.00 (247.00, 392.00)	−3.926	0.000**
β-HCG, mIU/mL	0.93 (0.22, 7.50)	0.12 (0.10, 0.20)	−3.482	0.000**
L_length, mm	46.57 (35.22, 56.93)	68.52 (55.29, 121.35)	−3.515	0.000**
L_ short diameter, mm	36.19 (30.90, 43.23)	48.91 (35.82, 61.37)	−2.408	0.016*
CT attenuation of Lesion, HU				
L_ctp	43.24 ± 5.86	32.16 ± 7.74	4.887	0.000**
L_cta	54.20 ± 8.57	44.58 ± 12.28	3.062	0.004**
L_ctv	63.23 ± 9.54	54.17 ± 12.45	2.709	0.010**

Unless indicated otherwise, the data presented are the number of lesions, with percentages in parentheses. *p < 0.05; **p < 0.01. Variables with normal distribution (Kolmogorov–Smirnov test) are presented as mean ± SD; SD, standard deviation. Variables with non-normal distribution are presented as median [interquartile range (IQR)].

AFP, alpha-fetoprotein (0–2 ng/mL); CEA, carcinoembryonic antigen (0–5 ng/mL); CA125, carbohydrate antigen 125 (0–35 U/mL); CA19-9, carbohydrate antigen 19-9 (0–37 U/mL); LDH, lactate dehydrogenase (100–300 U/L); β-HCG, β-human chorionic gonadotropin (0–5 mIU/mL); L_length, lesion length; L_short diameter, lesion short diameter; CT, computed tomography; L_ctp, CT attenuation of lesion unenhanced phase; L_cta, CT attenuation of lesion arterial phase; L_ctv, CT attenuation of lesion venous phase.

### CT qualitative parameters

As shown in **Table 2**, there were statistically significant differences between the testicular ERMS group and the testicular seminoma group in terms of whether the lesions were parallel to the ipsilateral inguinal region [parallel relationship (L & IIR)], the vascular ball sign, the components of the lesions (solid and cystic-solid), abnormal widening of the spermatic veins, the abnormality of blood vessels within the lesion, and cryptorchidism (all *p* < 0.05). **Table 2** summarizes the CT qualitative parameters between the two groups of testicular ERMS and testicular seminoma. The consistency results among the observers regarding the qualitative data can be found in the **Supplementary Material**.

**Table 2:** CT qualitative parameters.

Parameters	All (*n* = 45)	Testicular seminoma (*n* = 30)	Testicular ERMS (*n* = 15)	*χ* ^2^	*P*
Lesion site, *n* (%)					
Left	19 (42.22)	13 (43.33)	6 (40.00)	0.046	0.831
Right	26 (57.78)	17 (56.67)	9 (60.00)		
Parallel relationship (L & IIR), *n* (%)					
No	24 (53.33)	23 (76.67)	1 (6.67)	19.688	0.000**
Yes	21 (46.67)	7 (23.33)	14 (93.33)		
Vascular ball sign, *n* (%)					
No	19 (42.22)	5 (16.67)	14 (93.33)	24.094	0.000**
Yes	26 (57.78)	25 (83.33)	1 (6.67)		
Abnormal widening of the spermatic veins, *n* (%)					
No	14 (31.11)	4 (13.33)	10 (66.67)	13.272	0.000**
Yes	31 (68.89)	26 (86.67)	5 (33.33)		
Disease components, *n* (%)					
Solid	38 (84.44)	29 (96.67)	9 (60.00)	10.235	0.001**
cystic-solid	7 (15.56)	1 (3.33)	6 (40.00)		
Abnormal dilation of the testicular artery, *n* (%)					
No	22 (48.89)	15 (50.00)	7 (46.67)	0.044	0.833
Yes	23 (51.11)	15 (50.00)	8 (53.33)		
Lymph node metastasis, *n* (%)					
No	27 (60.00)	21 (70.00)	6 (40.00)	3.750	0.053
Yes	18 (40.00)	9 (30.00)	9 (60.00)		
Internal necrosis within the lesion, *n* (%)					
No	35 (77.78)	21 (70.00)	14 (93.33)	3.150	0.076
Yes	10 (22.22)	9 (30.00)	1 (6.67)		
Abnormality of blood vessels within the lesion, *n* (%)					
No	26 (57.78)	25 (83.33)	1 (6.67)	24.094	0.000**
Yes	19 (42.22)	5 (16.67)	14 (93.33)		
Cryptorchidism, *n* (%)					
No	33 (73.33)	18 (60.00)	15 (100.00)	8.182	0.004**
Yes	12 (26.67)	12 (40.00)	0 (0.00)		

Unless indicated otherwise, the data presented are the number of lesions, with percentages in parentheses. * *p* < 0.05; ** *p* < 0.01. Parallel relationship (L & IIR) = the parallel relationship between the lesion and the ipsilateral inguinal region.

### Differential diagnostic value of CT parameters in testicular ERMS and testicular seminoma

**Figure 1:**
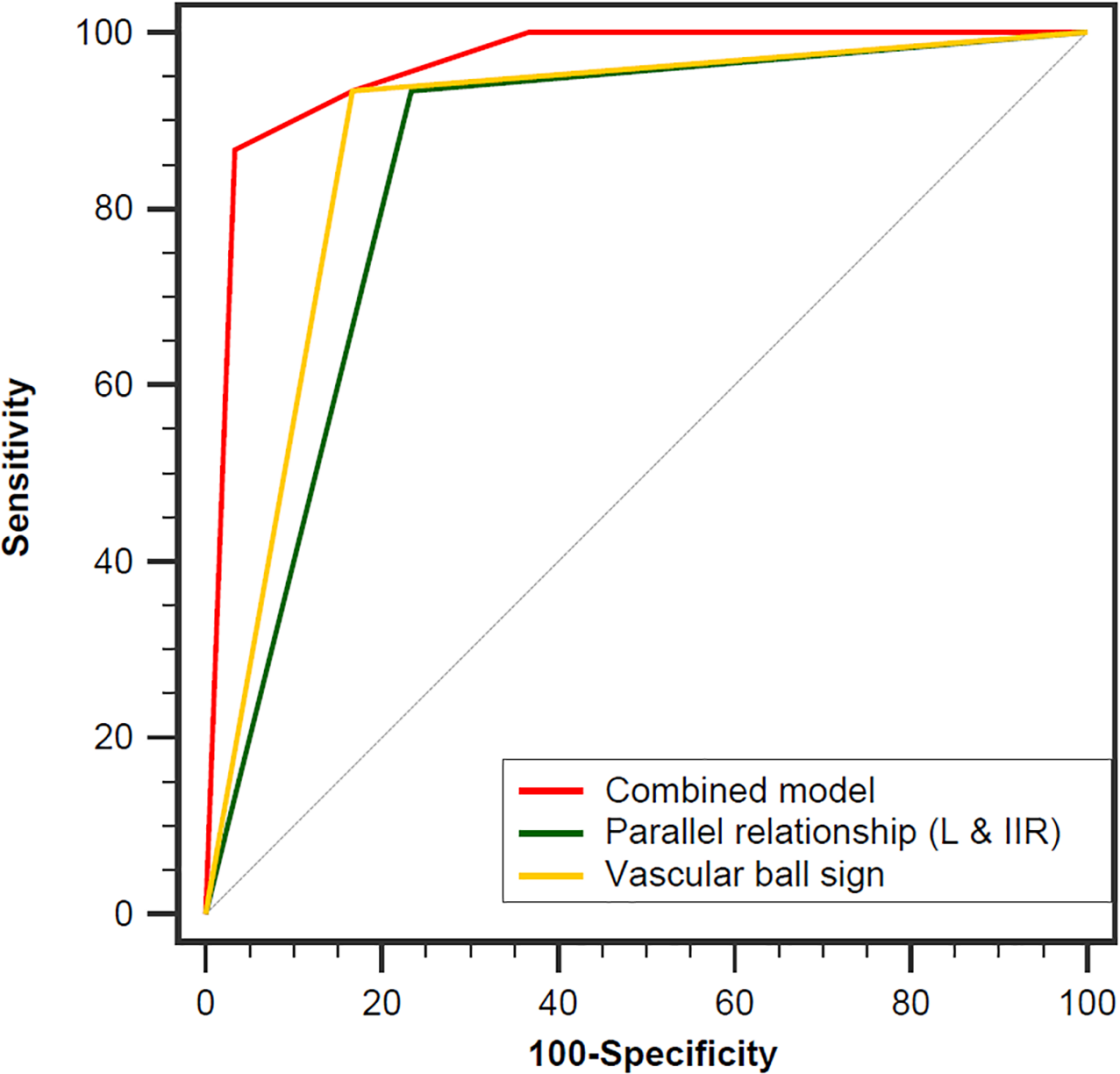
ROC curves of the combined model and other independent factors in the differential diagnosis of testicular ERMS and testicular seminoma. The AUC of the combined model was higher than that of the other two parameters (0.961 vs. 0.850 and 0.883, *p* < 0.05). ROC, receiver operating characteristic; AUC, area under the ROC curve; ERMS, embryonal rhabdomyosarcoma.

**Figure 2:**
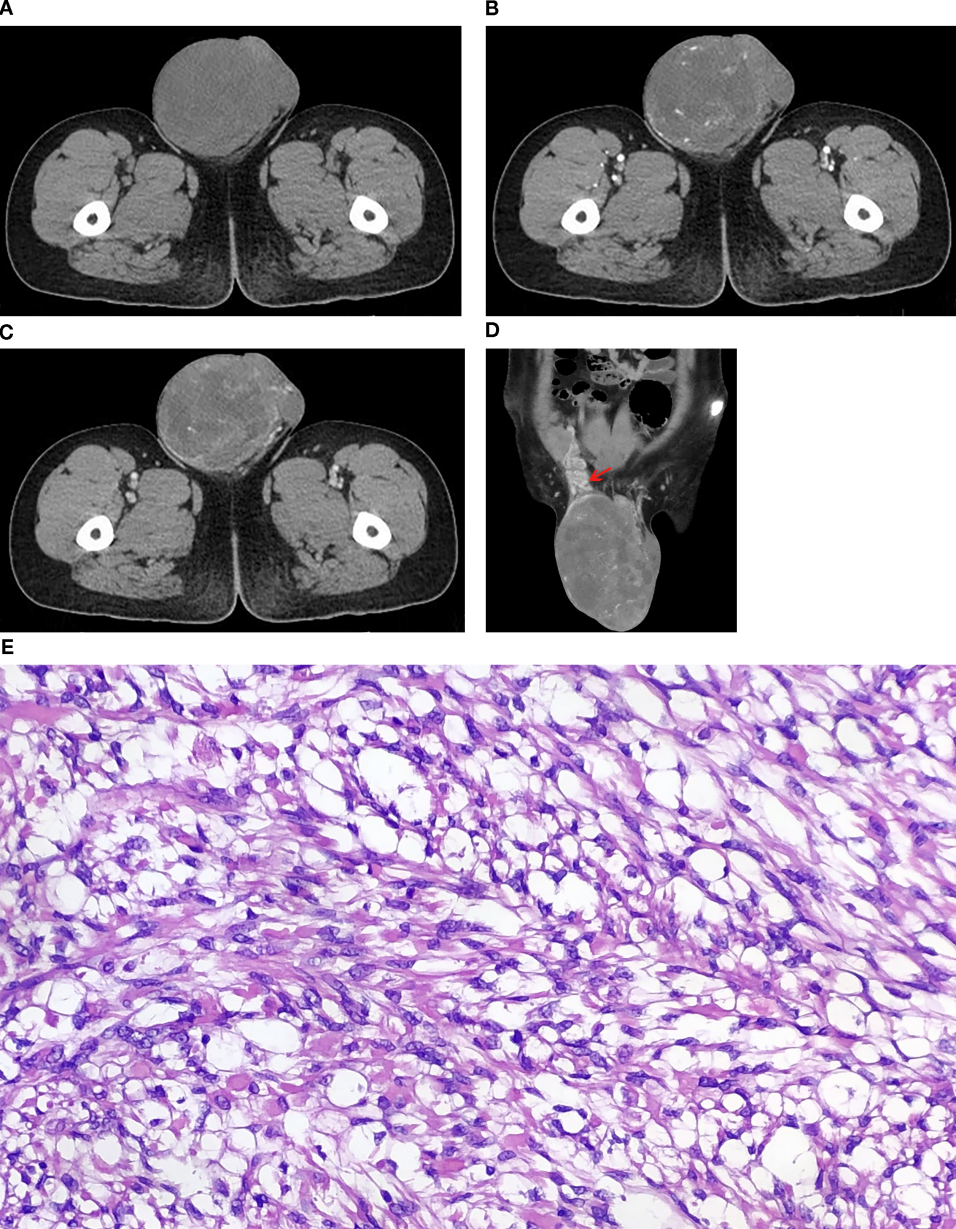
A 15-year-old male patient with right-sided testicular ERMS. **(A)** Non-enhanced CT image shows a soft tissue lesion in the right testicle, with uneven density within. **(B, C)** The arterial phase and venous phase show abnormality of blood vessels within the lesion. **(D)** The coronal view of venous phase shows that the spermatic veins are abnormally dilated (red arrow), and the coronal view shows that the long axis of the lesion is parallel to the ipsilateral inguinal region. **(E)** The pathological image shows embryonal rhabdomyosarcoma in the testis (H&E, ×200). ERMS, embryonal rhabdomyosarcoma.

**Figure 3:**
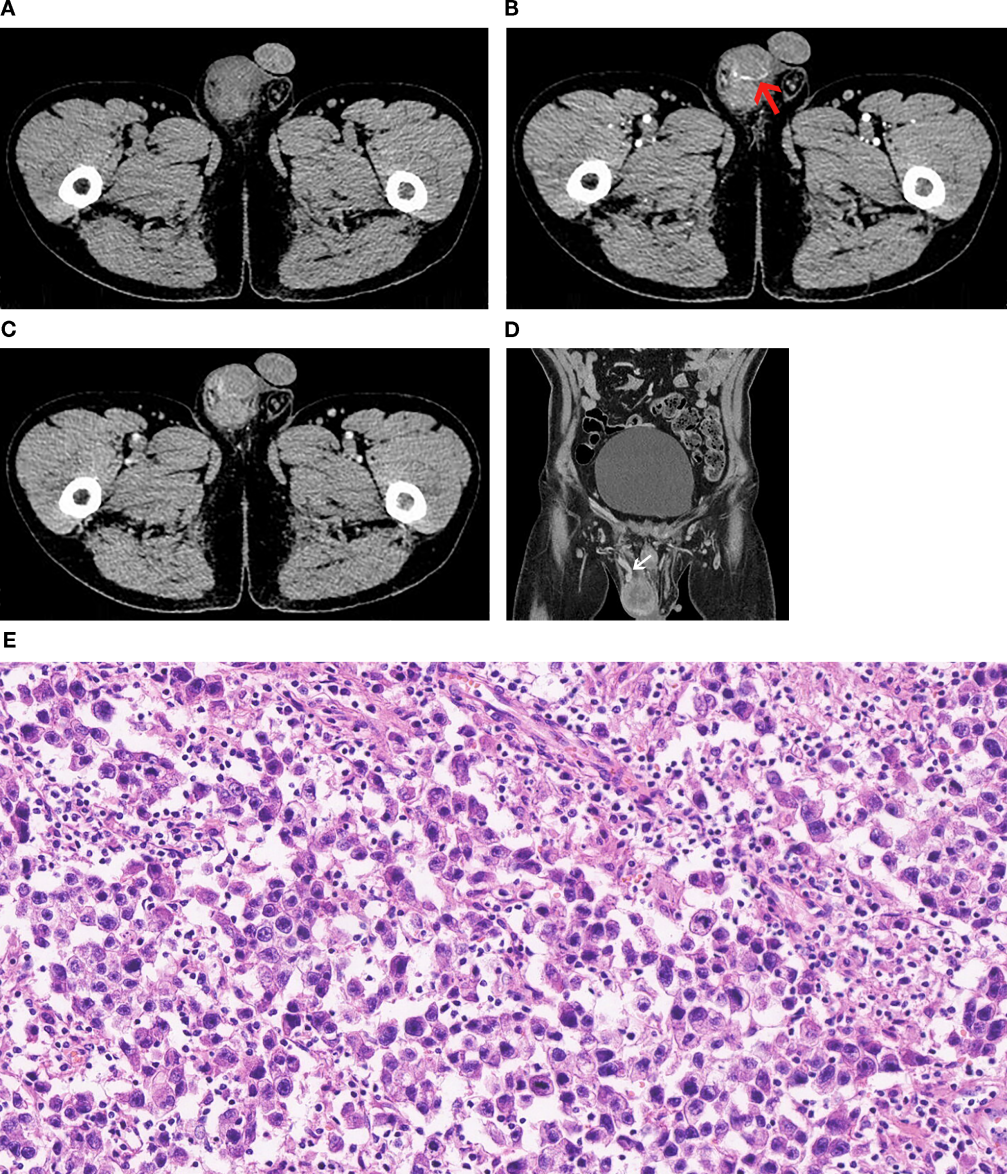
A 46-year-old male patient with right-sided testicular seminoma. **(A)** Non-enhanced CT image shows a soft tissue lesion in the right testicle. **(B, C)** The arterial phase and venous phase show the vascular ball sign (red arrow). **(D)** The coronal view shows that the spermatic veins are abnormally dilated (white arrow), and the long axis of the lesion is not parallel to the ipsilateral inguinal region. **(E)** The pathological image shows testicular seminoma (H&E, ×100).

Further analysis of the statistically significant parameters was conducted. Through univariate logistic regression, the results showed that the parallel relationship (L & IIR) and the vascular ball sign were independent factors for differentiating testicular ERMS from testicular seminoma. The above independent predictive factors were used to construct a model through multivariate logistic regression. The final constructed combined model demonstrated excellent diagnostic performance in differentiating testicular ERMS from testicular seminoma (AUC, 0.961). After internal validation using bootstrap resampling (1,000 iterations), the bootstrap-corrected AUC was 0.948, indicating good model stability and minimal overfitting. The valuable CT parameters for the differential diagnosis of testicular ERMS and testicular seminoma, as well as the AUC, sensitivity, and specificity values of the combined model, are shown in **Table 3**. DeLong’s test showed that the combined model was superior to the other parameters (*p* < 0.05). The ROC curve of the combined model is shown in **Figure 1**. Furthermore, the final established combined model is illustrated in **Figures 2** and **3**.

**Table 3:** Comparison of CT parameters for distinguishing testicular ERMS and testicular seminoma.

Model	Sensitivity	Specificity	AUC (95% CI)	Youden’s index
Parallel relationship (L & IIR)	93.3%	76.7%	0.850 (0.712–0.939)	0.700
Vascular ball sign	93.3%	83.3%	0.883 (0.753–0.960)	0.767
Combined model	86.7%	96.7%	0.961 (0.857–0.996)	0.833

Parallel relationship (L & IIR) = the parallel relationship between the lesion and the ipsilateral inguinal region.

Furthermore, follow-up examinations were conducted on the patients after the surgery. Follow-up duration: the median follow-up was 13 months (range, 2–122 months) for ERMS and 13.5 months (range, 2–84 months) for seminoma. The results showed that in the group with testicular ERMS, 10 out of 15 lesions showed progression (10/15, 66.7%; five cases of recurrence and five cases of distant metastasis), and the median time to progression was 7 months (range, 2–16.5 months); while in the group with testicular seminoma, only one case showed disease progression (1/30, 3.3%; recurrence).

## Discussion

Testicular ERMS is a rare and aggressive mesenchymal neoplasm. In this study, 15 cases of intratesticular ERMS and 30 cases of testicular seminoma were included. Based on the analysis of CT-enhanced images, the preliminary results showed that there were differences in multiple parameters between the two groups. Further single and multiple factor analyses revealed that the parallel relationship (L & IIR) and the vascular ball sign were independent predictors for differentiating the two types of neoplasms. Using this to establish a combined model, the results showed that the established combined model had high diagnostic efficacy, reaching 0.961. Further follow-up of the patients after surgery showed that the probability of disease progression in the testicular ERMS group was significantly higher than that in the testicular seminoma group.

### Clinical characteristics

RMS mainly occurs in the head and neck, urogenital tract, and limbs (15). RMS of organs adjacent to the bladder and testicles accounts for many of these neoplasms located in the urogenital tract system (15). Although paratesticular ERMS is more common, intratesticular ERMS does exist. Furthermore, it is a highly invasive malignant neoplasm (5, 16). Therefore, early diagnosis and treatment can improve the survival of patients. Testicular seminoma, as the most common malignant neoplasm of the testicles, does not have significant differences in symptoms between the two and usually presents as painless testicular masses, with a hard texture on palpation (6, 15). The literature indicates that testicular ERMS is more common in children and young adults, while testicular seminoma is more prevalent in patients 30–50 years old (1, 9, 10). The results of this study show that the median age of testicular ERMS cases was 13 years, while the median age of testicular seminoma was 32 years. Previous studies have suggested that the age of patients diagnosed with seminoma is 10 years older than that of patients with non-seminoma neoplasms (9, 10). Therefore, the difference in age distribution between the two also has certain diagnostic value. Testicular ERMS, as a type of non-seminoma, the results of this study are consistent with previous research findings. The American Society of Clinical Oncology (ASCO) has identified three key serum markers for testicular seminoma: AFP, β-HCG, and LDH (17). The key serum markers reported in previous literature for testicular ERMS are also these three (18). However, the literature indicates that most cases fall within the normal range, especially in testicular ERMS (17, 18). The study suggests that an elevated LDH level indicates a stronger invasive ability of the neoplasm. The results of this study show that although there are differences in AFP, CA125, CA19-9, and β-HCG between the two groups, all are within the normal range. The serum LDH level of testicular ERMS is higher than that of testicular seminoma, which is consistent with previous research findings. Testicular ERMS has a higher degree of malignancy and is more invasive.

### CT parameters

Imaging examinations are of vital importance for assessing the size of the primary neoplasm, local and distant metastases, long-term patient follow-up, and early detection of recurrence. Previous literature has indicated that the median length of testicular ERMS is 75 mm, while the length of testicular seminoma is typically 30 to 50 mm (15, 19, 20). This study compared the conventional CT parameters of the two groups of lesions. The results showed that the length and short diameter of the lesions in the testicular ERMS group were greater than those in the testicular seminoma group, and the difference was statistically significant. The results also confirmed previous studies. Due to the differences in neoplasm components and the presence of the vascular barrier, the enhancement of testicular ERMS is not as obvious as that of testicular seminoma. Meanwhile, previous literature reports have indicated the presence of cystic or necrotic lesions in testicular ERMS and the vascular disorder within the neoplasm (21). The results of this study also confirm the reports in the literature. Testicular seminoma may cause abnormal thickening of the spermatic veins due to neoplasm compression or invasion. The results of this study also confirm that the incidence of spermatic vein dilation in spermatocytic tumors is higher (86.7%), which is consistent with previous research findings (20). During the growth process of testicular ERMS neoplasms, a large number of blood vessels are required to support the proliferation and metabolism of neoplasm cells, resulting in the thickening of the testicular arteries. At the same time, the neoplasm may also secrete angiogenic factors, further promoting the growth and dilation of blood vessels. However, the thickening of the testicular arteries can also be observed in cases of testicular inflammation, varicocele of the spermatic vein, and other testicular neoplasms. The results of this study indicate that the abnormal dilation of the spermatic vein and the vascular ball sign in testicular seminoma have a higher occurrence rate. This may be due to the more uniform internal composition of testicular seminoma, which presents as a uniform soft tissue density. Therefore, the blood vessels are mostly distributed at the edge of the mass, forming a “vascular ball sign” (19). Some scholars have reached the same conclusion as us, that is, the presence of blood vessels surrounding testicular spermatocytic tumors is a characteristic manifestation of testicular spermatocytic tumors (19, 20). ERMS is more often characterized by cystic-solid components (40%) and the abnormality of blood vessels within the lesion (93.3%), which is consistent with its highly invasive, necrotic, and angiogenic features (15, 18). Furthermore, according to previous studies, cryptorchidism is regarded as a major risk factor for the development of testicular seminoma, and testicular seminoma is prone to metastasis to the retroperitoneum (6). The results of this study show that among patients with testicular seminoma, 40.0% of the cases have a history of cryptorchidism. The vascular ball sign has a good diagnostic efficacy in differentiating testicular ERMS from testicular seminoma, with an AUC of 0.883 (95% CI 0.753–0.960). It is worth noting that in this study, CT images were observed through three-dimensional reconstruction, and it was found that the long diameter of testicular ERMS neoplasms was parallel to the ipsilateral inguinal region. Among the 15 lesions, 93.33% had a parallel relationship (L & IIR). The parallel relationship likely reflects invasive growth along the spermatic cord fascial plane, which is an anatomical pathway of spread for embryonal rhabdomyosarcoma and the differential diagnosis of testicular ERMS and testicular seminoma (AUC, 0.850). In the identification of testicular ERMS and testicular seminoma, the combined model established by integrating the above two qualitative CT parameters achieved the best diagnostic efficacy (with an AUC of 0.961), having a sensitivity of 86.7% and a specificity of 96.7%. Internal validation was performed using bootstrap resampling with 1,000 iterations, and the bootstrap-corrected AUC was 0.948, confirming satisfactory model stability and low risk of overfitting.

Furthermore, the median follow-up period was relatively short (13 months), which may have underestimated the occurrence of late recurrence, especially for seminoma. However, the progression rate of ERMS was significantly higher than that of other types (66.7% vs. 3.3%), which is consistent with the well-known aggressive biological characteristics of this tumor (15, 18) and also confirms the accuracy of our comparison results. The parallel relationship proposed in this study further reflects the aggressive growth pattern of ERMS and is consistent with our follow-up results.

This study also has certain limitations. First, there is a problem of selection bias in the retrospective study, and single-center studies may have referral bias. Additionally, there was heterogeneity in CT equipment and scanning protocols during the 12-year study period in the retrospective study. Second, although 15 intratesticular ERMS lesions were included in this study for analysis, due to their rarity and the limited information from previous literature, it was not possible to conduct a comprehensive analysis by combining other imaging examination methods, such as MRI and ultrasound. Only a preliminary exploration was carried out. Third, due to the rarity of the cases, only 15 cases of testicular ERMS were finally included in the study. Although the present study achieved a high diagnostic value (with an AUC of 0.961 and a bootstrap-corrected AUC of 0.948), further independent external validation with expanded sample sizes in single-center and multi-center studies is still warranted. Finally, due to the limited number of cases, although the follow-up study revealed a higher incidence of disease progression after testicular ERMS surgery, however, due to the limited number of cases, further prognostic analysis was not conducted. Therefore, it is necessary to further increase the sample size to deepen our understanding of this disease.

## Conclusion

In conclusion, the existence of a parallel relationship between the lesion and the ipsilateral inguinal region, as well as the vascular ball sign, is helpful in differentiating testicular ERMS from testicular seminoma. Early identification helps guide preoperative counseling, surgical planning, and early multidisciplinary treatment for ERMS, which has a high progression rate. Meanwhile, the recurrence or metastasis rate of testicular ERMS after surgery is higher. Close follow-up observation is necessary to improve the prognosis of patients.

## Data Availability

The original contributions presented in the study are included in the article/**Supplementary Material**. Further inquiries can be directed to the corresponding author.
