# Supplementary material for: Differential diagnosis of testicular embryonal rhabdomyosarcoma and testicular seminoma with enhanced CT: a retrospective study
Source: Front Oncol. 2026 Jun 3;16:1707887. doi: 10.3389/fonc.2026.1707887 (PMC13271905; doi:10.3389/fonc.2026.1707887)
Supplement: Supplementary file 1 [file DataSheet1.pdf]

Materials and Methods

Data collection

Cryptorchidism refers to a medical history of congenital undescended testis (unilateral/bilateral) confirmed by medical records and imaging, including both untreated cases and cases with orchidopexy in childhood.

Laboratory analysis of serological indicators was performed via routine blood tests within 1 week before treatment. Two experienced radiology diagnostic physicians with over 10 years of experience jointly reviewed the films and analyzed the CT images, who were blinded to the clinicopathological information, and the CT images were interpreted independently. The length of the lesion is measured by taking the measurement along the largest plane of the lesion. The short diameter was measured according to the maximal short diameter perpendicular to the length of the lesion maximum cross-sectional area. The physicians observed the CT images to determine if there were lesions on both sides and recorded whether the lesions were located on the left or right side of the testicle. At the same time, the CT attenuation of the lesion was measured at the maximum cross-sectional area (unenhanced phase, arterial phase and venous phase), avoiding necrotic areas. All the CT attenuation, short diameter and length of lesions were taken three times, and the mean value was calculated for the next statistical analysis.

In the event of a discrepancy in results, the decision is made by consultation between the two radiologists.

Image acquisition and analysis

Scanning equipment: Revolution CT/Revolution Apex CT (General Electric Company, GE), Brilliance iCT (Philips), Discovery CT HD750 (General Electric Company, GE), Somatom Definition Flash (Siemens), Somatom Force (Siemens).

The iodine contrast agent consisting of iohexol (350 mg I/ml, GE Medical Systems) and ioversol (320 mg I/ml, GE Medical Systems) was administered at a dosage of 1.5 mL per kilogram of body weight, with the injection rate of 3-3.5 mL per second (Adult dose) by a dual-head pump injector (Medrad, Warrendale, USA). For children, the dose is determined based on age and the drug instructions. Finally, 20 mL of saline flush was injected at a rate of 3 mL/s.

The examiner assumes the supine position, raises both arms to the side of the head, places the body in the center of the examination bed, and aligns the scanning horizontal line with the midaxillary line. Taking the diaphragm top as the baseline, the abdominal anteroposterior view is used as the positioning image. The scanning range is from the diaphragm top to 2 cm below the pubic symphysis (the abdomen (including the pelvic area)). If only pelvic CT scan, the scanning range is from the iliac crest to the 2 cm below the pubic symphysis. After completing the plain scan, using the prescribed flow rate, the high-pressure injector is used to inject the iodine contrast agent intravenously in a bolus manner, and perform dual-phase enhanced scans at 30 seconds and 70 seconds after injection.

The tube voltage for CT scanning is 120 kVp/kV. The automatic tube current or tube current ranges from 120 to 600 mA/mAs. The collimator width is 64 × 0.625 mm, 80 mm, and 128 × 0.6 mm. The tube rotation speed is 0.27-0.5 s/rotation. The pitch is

1.375, 1, and 0.984. The field of view is 500 × 500 mm or 600 × 600 mm. The matrix is 512 × 512. The reconstruction layer thickness is 0.625 mm, 1.25 mm, and 5 mm.

Two experienced radiology diagnostic physicians with over 10 years of experience jointly reviewed the films and analyzed the CT images, who were blinded to the clinicopathological information, and the CT images were interpreted independently. They collected and recorded the quantitative and qualitative parameters related to CT. The quantitative parameters of the CT features of the lesion, including its length, short diameter, the lesion site (left side, right side), the CT attenuation in the non-enhanced phase, arterial phase, and venous phase). The length of the lesion is measured by taking the measurement along the largest plane of the lesion. The short diameter was measured according to the maximal short diameter perpendicular to the length of the lesion maximum cross-sectional area. The physicians observed the CT images to determine if there were lesions on both sides, and recorded whether the lesions were located on the left or right side of the testicle. At the same time, the CT attenuation of the lesion was measured at the maximum cross-sectional area (unenhanced phase, arterial phase and venous phase), avoiding necrotic areas. The ROI (Region of Interest) was positioned as follow: (1) ROIs were placed on the maximum solid cross-sectional area of the lesion (the size of the ROI should occupy 1/2 to 2/3 of the largest slice) to avoid necrotic/cystic areas; (2) For lesions with extensive necrosis (>50% of the lesion area), ROIs were placed on the peripheral solid component (viable tumor tissue), at this time, the size of the ROI is 1/2 to 2/3 of the largest layer of the solid part; (3) the "three measurements" refer to three separate ROI placements on the same maximum solid cross-sectional slice

by the same radiologist, with the mean value used for analysis. All the CT attenuation, short diameter and lengthwere independently measured three times by two radiological diagnostic physicians. Then, the mean values of the two radiological diagnostic physicians were calculated for next statistical analysis.

The qualitative parameters of the CT features of the lesion, including the lesion site (left side, right side), the parallel relationship between the lesion and the ipsilateral inguinal region, the vascular ball sign, abnormal widening of the spermatic veins, disease components (solid, cystic-solid), abnormal dilation of the testicular artery, lymph node metastasis situation, internal necrosis within the lesion, abnormality of blood vessels within the lesion, and cryptorchidism. By reconstructing the three-dimensional CT images, multiple perspectives can be observed from the coronal, sagittal and transverse positions.

By reconstructing the three-dimensional CT images, multiple perspectives can be observed from the coronal, sagittal and transverse positions. By observing the relationship between the lesion and the ipsilateral inguinal region in the coronal position, recording whether there is a parallel relationship. The"parallel relationship" is defined as the long axis of the intratesticular lesion forming an angle of 0-15° with the longitudinal axis of the ipsilateral inguinal canal on coronal 3D reconstructed CT image. Observe the condition of the blood vessels around the lesion. Check if there is any thickening and disorder of the blood vessels within the mass, or if the blood vessels are clustered together around the mass (the vascular ball sign). Observe the condition of the blood vessels around the lesion, and check if there are any thickening and disorder

of the blood vessels within the mass, or if the blood vessels are clustered together around the mass (the vascular ball sign). Combined with other imaging data, such as ultrasound, observe and record the state of the spermatic vein (whether it is thickened), the nature of the lesion (solid, cystic-solid), and the state of the testicular artery (whether it is thickened). To assess whether the lymph nodes are abnormal, if the short diameter of the lymph nodes is greater than 10mm, it is considered that there is lymph node metastasis. By combining the enhanced images, it is possible to determine whether there is necrosis within the mass. At the same time, review the medical history and assess the imaging data to determine whether the patient has undescended testicles.

For qualitative parameters, if there are differences in the results, then this decision will be made after a joint discussion by two radiologists. The proportion of cases requiring consensus discussion was <5% (only 2 out of 45 cases, limited to the judgment of mild spermatic vein widening).

Follow-up protocol: Telephone follow-up was conducted at 3 months and 6 months after the surgery, and then the electronic medical record system was used for follow-up. Subsequently, follow-up was carried out once a year.

Treatment differences: all patients in both groups received radical orchiectomy as the primary treatment; ERMS patients received adjuvant chemotherapy (VAC regimen: vincristine + actinomycin D + cyclophosphamide; AVCP regimen: Adriamycin+Vincristine+Cyclophosphamide+Prednisone Regimen) per pediatric oncology guidelines, and seminoma patients received adjuvant chemotherapy (BEP regimen: bleomycin + etoposide + cisplatin) per ESMO guideline.
